# Fatty acid profiles from twenty-one plant species identified as palatable for pasture-raised laying hens

**DOI:** 10.1371/journal.pone.0336271

**Published:** 2025-11-14

**Authors:** Gonzalo J. Diaz, Yandy J. Aguillón-Páez

**Affiliations:** Laboratorio de Toxicología, Facultad de Medicina Veterinaria y de Zootecnia, Universidad Nacional de Colombia, Bogotá, Colombia; ICAR - Indian Agricultural Research Institute, INDIA

## Abstract

A study was conducted to determine the fatty acid (FA) profiles of 21 plants voluntarily eaten by pasture-raised hens. Samples were collected from a very humid premontane forest during two dry and two rainy seasons within a year. FA were analyzed as methyl esters using a gas chromatograph coupled with a flame ionization detector. In general, no major changes in total saturated (SFAs), monounsaturated (MUFAs) or polyunsaturated FA (PUFAs) were observed within each plant among the four sampling times. However, for seven plants, large differences (3 times as much) in MUFAs content were observed and for *Heliconia sp*. the PUFAs content in the January sampling was almost double than the one found in July (43.3 vs 22.2%). On the other hand, large interspecies differences were observed in FA composition; for instance, *Bidens rubifolia* was predominantly PUFA-rich while *Musa paradisiaca* and *Heliconia* *sp*. were predominantly SFA-rich. The predominant FA in 86% of the plants were the PUFAs ɑ-linolenic acid (ALA, omega-3) and linoleic acid (LA, omega-6), and in almost all plants, the omega-6 content was lower than the omega-3 content. Therefore, the omega-6 to omega-3 FA ratio in all plants ranged from 0.1 to 1.1, which is an ideal ratio to improve the n-6/n-3 ratio of egg yolks. Interestingly, maize leaves were found to contain a high percentage of ALA and n-6/n-3 ratios ranging from 0.1 to 0.2 and could therefore be considered in pasture-raised laying hens. The results of the present study indicate that grazing plants can be a sustainable alternative source of n-3 FA for laying hens, in replacement of expensive marine sources.

## Introduction

Fatty acids (FA) are the most abundant form of reduced carbon chains available in nature and have diverse uses ranging from food to industrial raw materials [1]. FA are usually linear monocarboxylic chains of variable length: short-chain FA (2–6 carbon atoms), medium-chain FA (6–12 carbon atoms), long-chain FA (14–18 carbon atoms), and very long-chain FA (20–24 carbon atoms, derived from 18-carbon molecules). According to the number of double bonds, they can be divided into saturated (no double bonds), mono-unsaturated (one double bond), and polyunsaturated (two or more double bonds). In plants, FA are components of glycerol-containing lipids or glycerolipids, sphingolipids and extracellular lipids (cuticular waxes and lipid polyesters) [2]. All are involved in energetic, metabolic, and structural activities. Plants represent an important renewable source of FA because many species accumulate them in the form of triacylglycerols, which are major storage components in seeds. Furthermore, plants differ from many eukaryotes, including yeasts and mammals, in that they can produce a large diversity of FA structures that can differ with respect to carbon chain lengths, numbers and positions of double bonds, and carbon chain modifications such as hydroxylation [2].

Although more than 450 different FA are known in the plant kingdom, only five are widely found in membrane lipids, including phospholipids, galactolipids, and triacyl-glycerols: palmitic acid (16:0), stearic acid (18:0), oleic acid (18:1Δ9), linoleic acid (LA, 18:2 Δ 9,12), and α-linolenic acid (ALA, 18:3 Δ 9, 12,15) [3]. Long-chain FA are synthesized de novo from small precursors ultimately derived from photosynthetic precursors. Two enzyme systems, acetyl-CoA carboxylase and fatty acid synthase, are used. The end products of this synthesis are usually the saturated FA palmitate and stearate, with the latter predominating. Once the long-chain acids are formed, they can be elongated and desaturated [4].

The n-3 series of unsaturated FA is present mainly in fish oils (cod, salmon, sardine, sole, etc.), while the n-6 series is more common in vegetable oils (soybean, corn, sunflower, rape seed, etc.) [5]. However, leaf lipids in plants usually contain large proportions of ALA, which is an important component of the polar lipids of the chloroplast membrane. Further, wild leafy plants contain more ALA and less LA, while cultivated plants and seeds contain more LA, except for flax (*Linum usitatissimum*) [6].

Few studies have investigated the FA profile from wild leafy plants that are palatable for chickens. The objective of the present study was to characterize the FA profiles of twenty-one plant species identified as palatable for pasture-raised hens, collected from a very humid premontane forest in Colombia, both during the dry and the rainy seasons.

## Materials and Methods

### Samples

**Fig 1:**
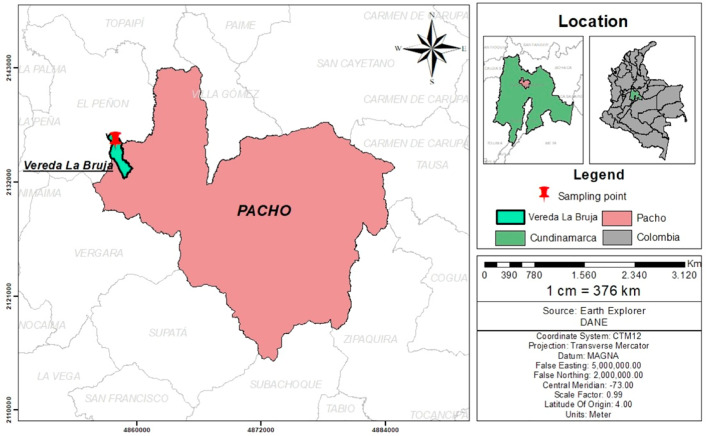
Geographical localization of the poultry farm where the sampling of the 21 plant species was carried out.

Sampling was carried out at a poultry farm located in the county “La Bruja”, city of Pacho, Cundinamarca, Colombia (5°07′50″ North; 74°09′30″ West) (Fig 1). The area corresponds to a very humid premontane forest with a mean altitude of 1314 m and temperatures ranging from 14 to 25°C all year long. All plants that were voluntarily eaten by free-range laying hens were collected for a total of 21 species. From each plant, a specimen was collected and sent for botanical classification to the Forest Herbarium “Gilberto Emilio Mahecha Vega”, Facultad del Medio Ambiente y Recursos Naturales of the Universidad Distrital “Francisco José de Caldas” in Bogota, Colombia. The species included in the study, their botanical families, Spanish common names, and phenological stage when collected are shown in Table 1. All animal experimental procedures conducted in this study comply with the standards and regulations established by the Ethics Committee of the National University of Colombia (Approval CB-FMVZ-UN-001–2022).

**Table 1:** Scientific name, botanical family, common name (in Spanish), phenological state and origin of the collected plants.

Scientific name	Family	Common name	Phenological state*	Origin**
*Trichanthera gigantea (Humbo & Bonpl)*	Acanthaceae	Nacedero	M	N
*Xanthosoma sagittifolium (L.) Schott*	Araceae	Bore	Vd	E/C
*Bidens rubifolia Kunth*	Asteraceae	Chipaca	B	N
*Melanthera nivea (L.) Small*	Asteraceae	Tamo-tamo	Vd	N
*Tithonia diversifolia (Hemsl.) A. Gray*	Asteraceae	Botón de oro	B	E/C
*Sechium edule (Jacq.) Sw.*	Cucurbitaceae	Guatila	Vd	E/C
*Acalypha diversifolia Jacq.*	Euphorbiaceae	Suan	Vd	N
*Acalypha macrostachya Müll. Arg.*	Euphorbiaceae	Lechero	Vd	N
*Desmodium cajanifolium (Kunth) DC.*	Fabaceae	Varilla	B	N
*Desmodium sp.*	Fabaceae	Pega-pega	B	N
*Heliconia sp.*	Heliconiaceae	Platanillo	I	N
*Malachra rudis Benth.*	Malvaceae	Malva	B	N
*Sida poeppigiana (K. Schum.) Fryxell*	Malvaceae	Escoba	B	N
*Musa paradisiaca L.*	Musaceae	Plátano	I	E/C
*Axonopus scoparius (Flüggé) Kuhlm.*	Poaceae	Micay	M	N
*Oplismenus burmannii (Retz.) P. Beauv.*	Poaceae	Grama	M	N
*Saccharum officinarum L.*	Poaceae	Caña de azúcar	Vd	E/C
*Zea mays*	Poaceae	Maíz	Vd	E/C
*Solanum nigrescens M. Martens & Galeotti*	Solanaceae	Yerbamora	B	N
*Myriocarpa stipitata Benth.*	Urticaceae	Agüachente	Vd	N
*Lantana camara L.*	Verbenaceae	Venturosa	B	N

*B = Bloom, Vd = Vegetative development, M = Mature, I = In florescence.

**N = Native, E/C = Exotic/Cultivated.

For the FA analysis, a total of four samples per plant were collected as follows: two during the “rainy season” (November 2021 and April 2022) and two during the “dry season” (January 2022 and July 2022). Sampling times were selected according to the historical records of rain and drought for the region, as follows: for November and April, 253 and 215 mm of precipitation, respectively; for January and July, 98 and 51 mm of precipitation, respectively. For the sampling procedure, approximately 500 g of leaves were collected from each plant, placed on newspaper, and taken to the laboratory (Laboratorio de Toxicología of the Universidad Nacional de Colombia) for the determination and quantitation of FA as described below. All analyses were carried out in duplicate and the results averaged.

### Extraction and derivatization of the fatty acids

The extraction of FA was performed according to a previously published method [7], with minor modifications. Table 2 summarizes the sample preparation procedure.

**Table 2:** Sample preparation for fatty acid profile determination in plant tissue (leaves).

1. Lyophilize or dry the samples at a temperature below 55°C.
2. Weigh 0.25 g of sample or the equivalent of 10–50 mg fatty acid content into a 16 mm x 125 mm test tube with a Teflon-coated polypropylene screw cap.
3. Add 3 mL of 10% HCl (hydrochloric acid) in methanol (prepared by slowly adding 10 mL of acetyl chloride to 100 mL anhydrous methanol) and 2 mL of toluene, stopper and vortex.
4. Heat at 90°C in a water bath for 2 h, without allowing the solvent to dry. Allow to cool to room temperature.
5. Carefully add 1 mL of hexane and 5 mL of 6% potassium carbonate (K_2_CO_3_) in water (dissolve 6 g of K_2_CO_3_ in 50 mL of water and make up to 100 mL) and vortex.
6. Centrifuge at 1500 x *g* (3000 rpm) for 5 min.
7. Transfer the organic phase (upper phase) to a 12 x 100 mm test tube with polypropylene screw cap.
8. Add 1 g of anhydrous sodium sulfate and 1 g of charcoal (only if the sample is colored).
9. Vortex and centrifuge at 1500 x *g* (3000 rpm) for 5 min.
10. Transfer the supernatant to an 11.5 x 76 mm test tube.
11. Filter through a 0.22 µm polytetrafluoroethylene (PTFE) syringe filter and collect into a 250 µL insert contained in a 1.5 mL autosampler amber vial.
12. Inject into the gas chromatograph.

### Chromatographic conditions

**Fig 2:**
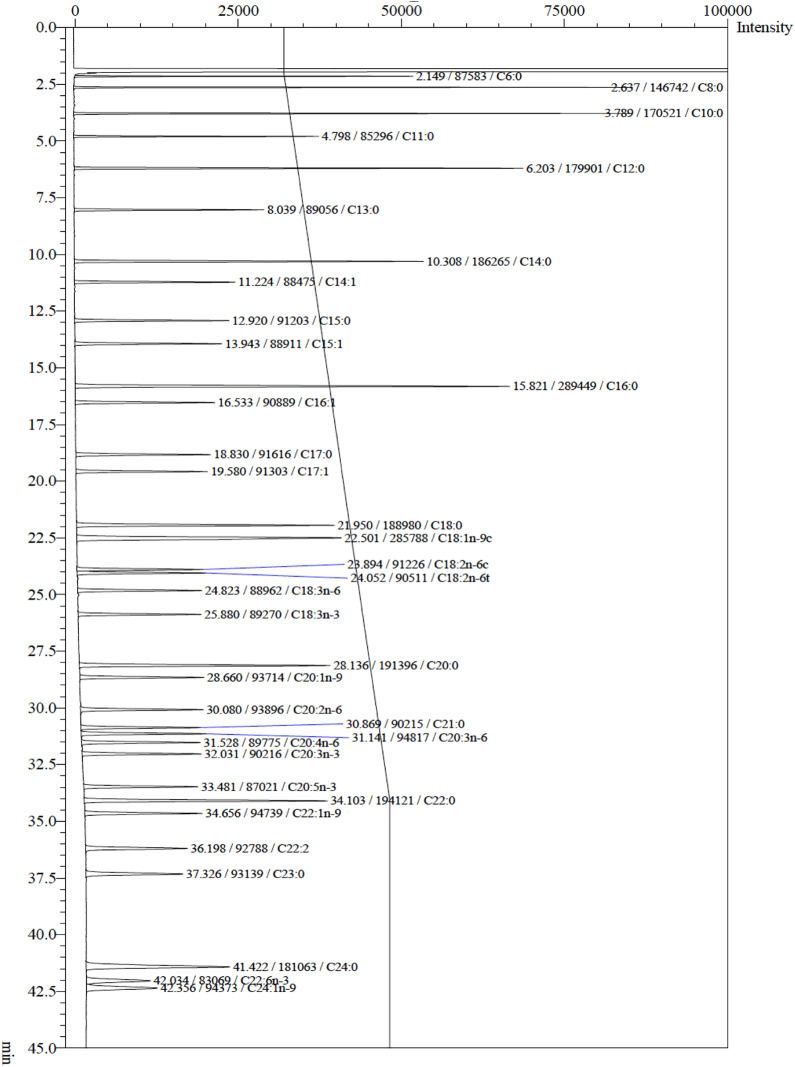
Chromatogram of the Supelco® 37 FAMES standard mixture, separated and detected under the chromatographic conditions described in “Materials and methods”.

The derivatized FA were analyzed as fatty acid methyl esters (FAMES) on a Shimadzu GC-2014A gas chromatograph (Shimadzu Scientific Instruments, Columbia, MD, USA), equipped with a flame ionization detector. FAMES were separated on a 30-m long capillary column with an inner diameter of 0.32 mm and a film thickness of 0.25 μm of bonded polyethylenglycol stationary phase (Supelco Omegawax 320 chromatographic column, Supelco, Inc., Bellefonte, PA, USA). Separation was obtained with a temperature ramp (initial temperature 140°C for 2 min followed by 2.5°C/min to 220°C and holding at 220°C for 10 min before returning to 140°C) using helium as the carrier gas and nitrogen as the make-up gas. The injection was in split mode with a split ratio of 1:20. The retention times were compared to those of known standards (37 FAME Mix, Supelco, Inc., Bellefonte, PA, USA) (Fig 2). Data were analyzed using the Shimadzu software Lab Solutions version 5.110.

### Preparation of the calibration standards

The working standard solution was prepared by quantitatively transferring 10 µL of the 37 FAMES solution to an autosampler vial to which 160 µL methylene chloride were then added. The Supelco® FAME mixture is prepared by weight and the weight percentage of each component is indicated in the standard insert. Each ampule contains 10 mg/mL of the FAME reference standard mix in methylene chloride. Therefore, the concentration of the working solution was 0.59 mg/mL of the total 37 FAMES. All FAMEs are present in the standard at a concentration of 2% by weight, except for C4:0, C6:0, C8:0, C10:0, C14:0, C18:0, C18:1n9c, C20:0, C22:0, and C24:0, which are present at 4% by weight and C16:0 which is present at 6% by weight.

Data were reported as the percentage of each FA present, normalizing the total amount of FAMES to 100%. The percentage of each component was calculated by dividing its area by the total sum of the peak areas and multiplying by 100.

### Statistics analysis

The data were analyzed using descriptive statistics, with results expressed as mean values for each forage species across the four sampling periods. Data processing and graphical visualization were performed using R statistical software (version 4.5.1, R Core Team) [8], with the tidyverse and ggplot packages. A circular bar chart with grouped data and color-coded seasons (November, January, April, and July) was used to visually compare seasonal trends. Additionally, a Principal Component Analysis (PCA) was conducted to reduce dimensionality and explore patterns of variation among plant species and sampling periods.

## Results

**Fig 3:**
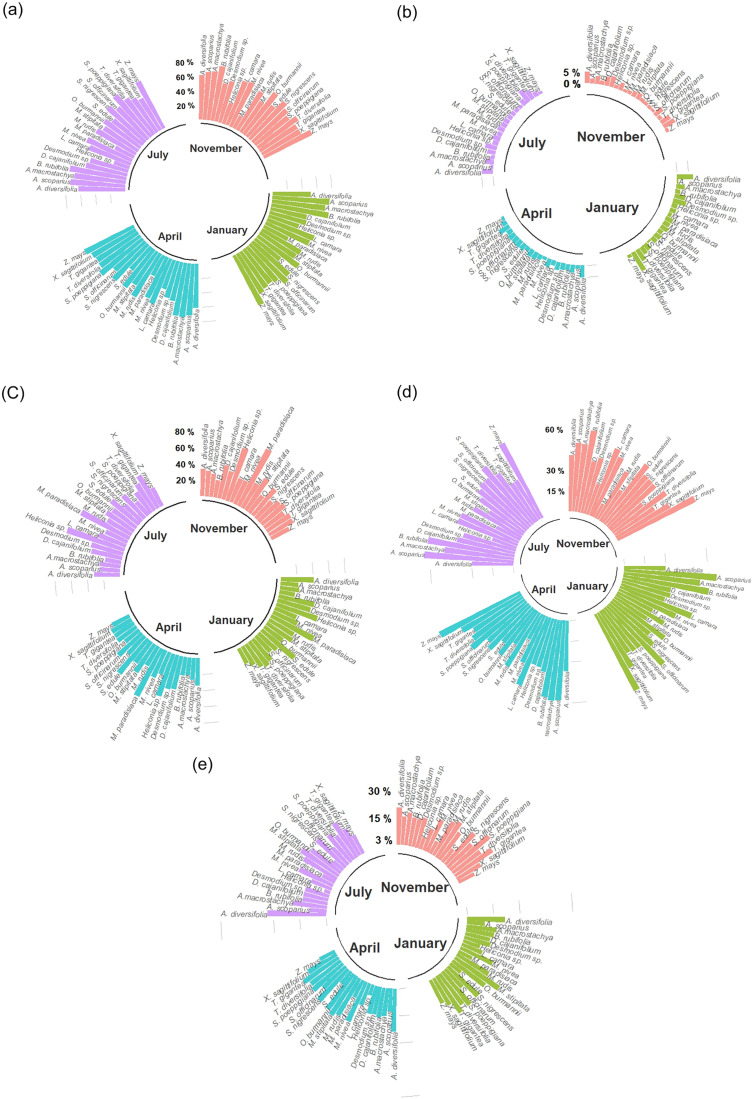
Total sum of polyunsaturated (a), monounsaturated (b), saturated (c) omega-3 (d) and omega-6 (e) fatty acids in 21 plants of a very humid premontane forest over four sampling periods (November, January, April, and July).

Fig 3 summarizes the total sum of polyunsaturated (PUFAs), monounsaturated (MUFAs), saturated (SFAs), omega-3 (n-3), and omega-6 (n-6) FA found in the plants analyzed in the present study. The SFAs found were C16:0, C17:0, C18:0, C20:0, C22:0, C23:0 and C24:0. The MUFAs found were C16:1, C18:1n9c and C18:1n7c, while the only PUFAs found were C18:2n6c and C18:3n3c.

Within each plant, no major differences in PUFAs content were observed among the four sampling times, except for *Heliconia sp*. (Fig 3a). For this plant the PUFAs content found in the November sampling was 30.3%, while in the January, April, and July samplings the values were 43.4, 42.4, and 22.2%, respectively. The Asteraceae *Bidens rubifolia* was the species with the highest PUFAs content, with an average of the four samplings of 76.8%. On the other hand, the plant species with the lowest PUFAs content was *Musa paradisiaca* with an average value 2.6 times lower than that of *Bidens rubifolia* (29.3%).

The MUFAs content in the plants tested was much lower than the PUFAs and SFAs content, with values ranging from 0.8 to 9.2% (Fig 3b). The lowest value (0.8%) was recorded for the grass *Axonopus scoparius* (January sampling) while the highest percentage (9.2%) was found in the Araceae *Xanthosoma sagittifolium*, also for the January sampling. Further, large differences in MUFAs content were observed among sampling times (up to 3-fold between samplings) for seven of the plants tested (*Axonopus scoparius*, *Musa paradisiaca*, *Sacharum officinarum*, *Sechium edule*, *Solanum nigrescens*, *Tithonia diversifolia*, and *Xanthosoma sagittifolium*). Also, large differences in MUFAs content were observed among the different plant species at different sampling times. For instance, in the November sampling, *Saccharum officinarum* and *Melanthera nivea* had both 1.3% MUFAs, whereas *Xanthosoma sagittifolium* and *Acalypha diversifolia* had 5.5 and 4.8%, respectively.

The SFAs content did not show major differences for any plant within the four sampling times; however, up to a 4-fold difference in SFAs content was found among different plant species, with values ranging from 19.3 to 78.7% (Fig 3c). The Asteracea *Bidens rubifolia* had the lowest average SFAs percentage compared to the other plants (21.1%). On the other hand, the highest average SFAs content was found in the Musaceae *Musa paradisiaca* (68.0%) and the Heliconiaceae *Heliconia sp*. (63.1%). In fact, *Musa paradisiaca* and *Heliconia sp*. showed the highest individual SFAs percentages corresponding to 78.7 and 75.2% in the November and July sampling times, respectively.

The total content of n-3 FA (which corresponded only to ALA) showed large differences among the different plant species analyzed (Fig 3d). On average, the lowest values were recorded for *Musa paradisiaca* (20.2%) and *Heliconia sp*. (28.0%). In contrast, three plant species had average n-3 percentages greater than 60%: the Poaceae plants *Axonopus scoparius* (60.2%) and *Zea mays* (63.4%) and the Asteraceae *Bidens rubifolia* (61.0%). In general, no major variations were observed in n-3 FA content among the different sampling times except for *Musa paradisiaca* and *Heliconia sp*. For *Musa paradisiaca* the n-3 FA percentage ranged from 11.9% (November sampling) to 28.6% (January sampling), whereas in *Heliconia sp*. the value ranged from 18.6% (July sampling) to 36.3% (April sampling).

In general, no major differences were found in n-6 FA content among the four sampling times within each plant (Fig 3e). However, one plant (*Musa paradisiaca*) showed a much lower n-6 FA content in the July sampling (3.6%) compared to the January sampling (10.1%). The lowest average n-6 FA percentages were found in *Heliconia sp*. and *Sechium edule*, with values of 6.6 and 8.2%, respectively. In contrast, the highest average values were those of the Euphorbiaceae *Acalypha diversifolia* (24.8%) and the Acanthaceae *Trichanthera gigantea* (24.2%).

**Fig 4:**
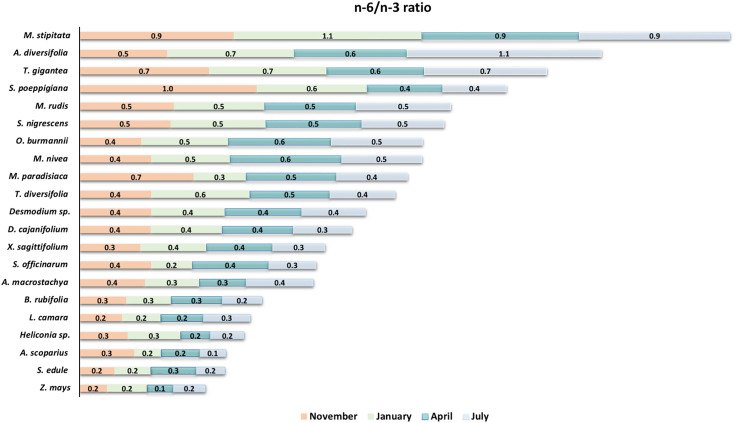
Omega-6 to omega-3 fatty acid ratio in the lipids extracted from 21 plants of a very humid premontane forest over four sampling periods.

Fig 4 shows the n-6/n-3 FA ratio of the 21 plants tested at the four different sampling times. Ratios were consistently <1.0 across nearly all species and seasons, with only 3 exceptions: *Sida poeppigiana* in the November sampling (ratio of 1.0), *Myriocarpa stipitata* in the January sampling (ratio of 1.1) and *Acalypha diversifolia* in the July sampling (ratio of 1.1).

**Fig 5:**
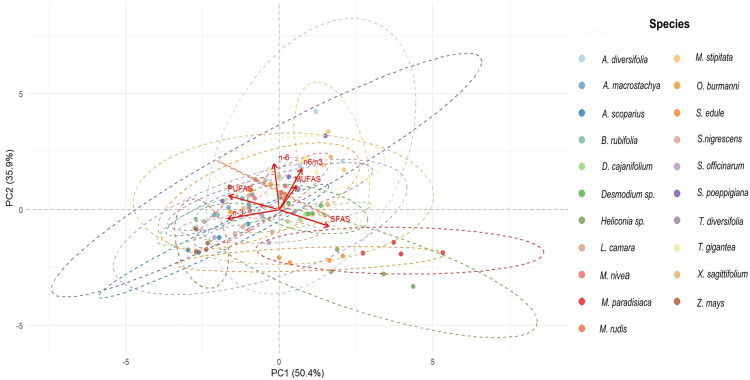
Principal component analysis (PCA) corresponding to the sum of fatty acids in 21 plant species evaluated at four different sampling times. SFAs: saturated fatty acids; MUFAs: monounsaturated fatty acids; PUFAs: polyunsaturated fatty acids; n-3: omega 3 fatty acids; n-6: omega 6 fatty acids; n-6/n-3: omega 6 to omega 3 fatty acid ratio.

The principal component analysis (PCA) of the SFAs, MUFAs, PUFAs, and total n-3 and n-6 FA is shown in Fig 5. PCA of the grouped FA showed a large variability in FA composition among the different species evaluated, with 86.3% of the total variation explained by the first two principal components (PC1 and PC2). PC1 (50.4%) was mainly associated with SFAs, while PC2 (35.9%) explained variability as a function of the unsaturated PUFAs, MUFAs, n-3 and n-6 FA content, as well as the n-6/n-3 ratio. The dispersion ellipses allowed the identification of groups according to lipid profile, highlighting species such as *Musa paradisiaca*, *Heliconia sp*. and *Sechium edule* with a strong association towards the saturated fraction; the remaining species presented a higher correlation with the unsaturated type variables.

## Discussion

Each cell in a plant must produce FA, and their synthesis must be finely controlled to balance the supply and demand of acyl chains. For most plant cells, this means matching the level of FA synthesis with membrane biogenesis and reconstruction. Depending on the stage of development, time of day, or growth rate, these requirements can vary greatly [9–10]. In addition to these variables, there are other biotic factors (viruses, bacteria, fungi, nematodes, and arthropods, among others) and abiotic factors (temperature (low or high), water (deficit or excess), ultraviolet rays, salt, and heavy metals) that can also affect FA synthesis [11]. Thus, the rates of FA biosynthesis in plants must be tightly regulated to accommodate all these variables [9–10].

Unsaturated fatty acids are important for membrane fluidity and their synthesis is regulated by two desaturases: FAD2 (fatty acid desaturase 2) and FAD3 (fatty acid desaturase 3) [12]. Studies have shown that cutting grass shoots reduces all FA by three to five times during the growing season, particularly ALA [13]. In the present study, similar PUFA values were observed within each plant at the four sampling times. In contrast, in a study that determined PUFAs in different Poaceae (*Bromus inermis*, *Beckmannia syzigachne*, *Elytrigia repens*, and *Carex atherodes*) during the summer, it was found that the PUFAs concentration was higher at the beginning of the summer compared to the end. In addition, it was reported that when the measurements were repeated after 24 and 72 hours, the content of PUFAs and MUFAs decreased [14]. In another trial conducted with *Cistus ladanifer*, it was found that there is a great variation in the composition of FA in the leaves throughout the seasons. For example, PUFAs were substituted by MUFAs during the warm seasons, suggesting an adaptive response to the environmental conditions [15]. Another possible explanation for the PUFAs content change over time is the phenological stage of the plant. Although a study conducted with Mediterranean herbs showed no significant effects of the phenological stage on the PUFAs content [16], other researchers claim that differences may exist during growth [17].

In the present study, oleic acid (C18:1 n-9 cis) was the predominant MUFA in all species with percentages that ranged from 0.68 to 9.21% (see supplementary data). In addition to the MUFAs content, the SFAs content also showed distinctive patterns among the studied plants. For example, the species *Musa paradisiaca* and *Heliconia sp*. had the highest SFAs values, with palmitic acid (C16:0) contributing to most to the total content (see supplementary data). This finding may be related to the species interaction with microbes; for example, obligate symbionts, such as arbuscular mycorrhizal fungi, which depend on the availability of plant-derived C16 FA for their own FA biosynthesis [18]. A study that evaluated the SFAs content in marrows and yellow zucchini leaves showed high percentages of SFAs: 36.73 and 33.15%, respectively [19]. However, these values were much lower than those found for *Musa paradisiaca* and *Heliconia sp*. in the present study (on average 68.0 and 63.1%, respectively). It has been established that exposure to cold is directly related to the level of fatty acid saturation [20–21]; however, this was probably not the case in the present study since the plants were not exposed to temperatures lower than 14°C. On the other hand, when evaluating the SFAs content in *Cistus ladanifer* leaves during different seasons over the course of a year, significant differences (p = 0.05) were found in winter, spring, summer, and autumn (10.4, 8.26, 8.29, and 12.0 mg/g dry matter, respectively) [15]. This contrasts however, with another trial [22], in which it was argued that the high temperature in the study area affected the concentration of palmitic acid (C16:0) in 12 forage species evaluated, since high temperatures cause a decrease in the concentration of ALA and an increase in the concentrations of palmitic (C16:0) and linoleic acids in plants. Apparently, the reduction of membrane fluidity (characterized by a greater incorporation of saturated fatty acids, primarily palmitic acid) is an adaptive mechanism that help to decrease the evapotranspiration in high-temperature environments [22].

Regarding the total n-3 FA content in the plants analyzed, it is noteworthy that the only n-3 FA found was ALA, with some plants containing ALA percentages above 60%. This finding suggests that some of the grazing plants analyzed can be an alternative source of n-3 FA for laying hens, in replacement of expensive marine sources. Studies conducted with other plant species showed that purslane (*Portulaca oleracea*) contained not only ALA, but also eicosapentaenoic acid (C20:5 n-3), docosahexaenoic acid (C22:6 n-3), and docosapentaenoic acid (C22:5 n-3) [17]. However, in a study conducted with several Australian varieties of *Portulaca oleracea*, the only n-3 FA detected was ALA, with percentages ranging from 58.0 to 62.5% [23]. In another study conducted in Suwon (South Korea), broccoli leaves showed a slightly higher percentage of ALA in fall compared to spring (52.52 vs. 48.81%, respectively) [24]; interestingly, these values were similar to those found in the present study, except for *Musa paradisiaca* and *Heliconia sp*., which had average ALA values of 20.2 and 28.0%, respectively.

Finally, in a study conducted in Russia to evaluate the effect of cutting and drying on lipids, FA, sterols, and systemic responses in leaves at three time points (24 h, 72 h, and new plant leaves) it was found that FA in fresh grass leaf tissues had a higher n-6/n-3 ratio than summer grasses [14]. In the present study, no differences were observed in the n-6/n-3 ratio among the four samples taken, even though the temperatures ranged from 14 and 25°C in the four sampling times. It is important to note that the study conducted in Russia is difficult to compare to that of a tropical country, given that Russia is a country with marked seasonal changes [14].

## Conclusions

The results of the present study show that the main FA present in the plants analyzed are palmitic acid, LA and ALA, with some plants having more than 60% ALA. The n-6 to n-3 FA ratio was below 1.0 in 86% of the plants, a very significant finding since the ideal ratio of n-6/n-3 from the human nutrition standpoint should be lower than 4–1 [25]. Interestingly, maize leaves, which do not have much use under normal conditions, were found to contain a high percentage of ALA and n-6/n-3 ratios ranging from 0.1 to 0.2 and could therefore be considered in pasture-raised laying hens. In general, no major changes in FA composition were found among the four sampling times for any of the 21 plants analyzed, a finding that might be related to the fact that ambient temperatures and the photoperiod do not change much in tropical areas close to the equator. This is a major advantage for farmers, as the nutritional value of the forage is reliable year-round. Interestingly, some plant species (e.g., *Bidens rubifolia*) were found to contain a much higher percentage of PUFAs compared to SFAs, whereas others (e.g., *Musa paradisiaca* and *Heliconia sp*.) had a much higher percentage of SFAs compared to PUFAs. In these plants, more than ¾ of the FA correspond to SFAs. Finally, the species collected and analyzed correspond to species that are voluntarily consumed by laying hens; these species can be used as a sustainable source of n-3 enrichment for egg yolks, given the importance of n-3 FA in human cardiovascular health. Further studies are needed to determine the potential use of the plants found to contain high levels of ALA in egg production. Also, it would be important to investigate the extent to which hens can transfer these FA to the egg yolk and how much plant material is needed to see a change in the FA profile of the eggs.

## Supporting information

S1 Fig Supporting information for Fig 3–5. Complete dataset of all fatty-acid methyl esters analyzed. This dataset was used to create Fig 3–5.
(XLSX)
